# The Factors Affecting Expansion of Reactive Tumor Infiltrating Lymphocytes (TIL) From Bladder Cancer and Potential Therapeutic Applications

**DOI:** 10.3389/fimmu.2021.628063

**Published:** 2021-02-25

**Authors:** Ahmet Murat Aydin, Brittany L. Bunch, Matthew Beatty, Ali Hajiran, Jasreman Dhillon, Amod A. Sarnaik, Shari Pilon-Thomas, Michael A. Poch

**Affiliations:** ^1^ Department of Genitourinary Oncology, Moffitt Cancer Center, Tampa, FL, United States; ^2^ Department of Immunology, Moffitt Cancer Center, Tampa, FL, United States; ^3^ Department of Pathology, Moffitt Cancer Center, Tampa, FL, United States; ^4^ Department of Cutaneous Oncology, Moffitt Cancer Center, Tampa, FL, United States

**Keywords:** adoptive cellular immunotherapy, Bacillus Calmette–Guerin, molecular subtypes, bladder cancer, tumor-infiltrating lymphocytes

## Abstract

Tumor infiltrating lymphocytes (TIL) therapy was shown to provide durable objective response in patients with metastatic melanoma. As a fundamental first step to bring TIL therapy to clinical use, identification of patients whose tumors yield optimal numbers of reactive TIL is indispensable. We have previously shown that expansion of tumor reactive TIL from primary bladder tumors and lymph node metastases is feasible. Here, we performed TIL harvesting from additional surgical specimens (additional 31 primary tumors and 10 lymph nodes) to generate a heterogenous cohort of 53 patients with bladder cancer (BC) to evaluate the tumor characteristics that lead to tumor-reactive TIL expansion. Among a total of 53 patients, overall TIL growth from tumor samples were 37/53 (69.8%) and overall anti-tumor reactive TIL were 26/35 (74.3%). Mixed urothelial carcinoma is associated with higher anti-tumor reactivity of expanded TIL than pure urothelial carcinoma (89.5% vs. 56.3%, p=0.049). The anti-tumor reactivity of expanded TIL from primary tumors previously treated with BCG immunotherapy were lower (33.3% vs. 82.6%, p=0.027) although T-cell phenotype (CD3+, CD4+, CD8+, and CD56+) was similar regardless prior of BCG therapy. Addition of agonistic 4-1BB antibody in culture media with IL-2 improved the number of expanded TIL from primary tumors previously treated with BCG immunotherapy. There was no significant difference between basal and luminal subtype tumors in terms of viable and reactive TIL growth. Our study demonstrates that TIL expansion is feasible across all BC patients and BC subtypes, and we suggest that TIL therapy can be a reasonable treatment strategy for various manifestations of BC.

## Introduction

Bladder cancer (BC) is the 10th most common form of cancer worldwide with a significantly high mortality rate in men (3.2 vs. 0.9, per 100.000) (1). In the US alone, the estimated number of new BC cases and deaths in 2019 were 80.470 and 17.670, respectively (2). Immunotherapy has been utilized for treatment of BC for more than four decades since the start of utilization of intravesical Bacillus Calmette-Guerin (BCG) for treatment of non-muscle invasive bladder cancer (NMIBC) (3). Nonetheless, about 20% to 45% of high-risk NMIBC progress to muscle-invasive bladder cancer (MIBC) despite endoscopic tumor resection and BCG immunotherapy (4). Moreover, about 20 to 40% of patients with MIBC experiences disease recurrence within 5 years of neoadjuvant chemotherapy (NAC) and radical cystectomy (RC) (5). Metastatic BC is a very lethal disease with an overall survival of approximately 12 months (6). In the past 5 years, immune checkpoint blockade (ICB) has been utilized for management of metastatic BC. Single agent checkpoint inhibitors provided objective responses in about only one fifth of the patients after first-line therapy (7). Therefore, there is still an unmet need of novel effective therapies for management of BC across all stages.

Adoptive cell therapy (ACT) has become a real prospect for treatment of solid tumors (8). TIL therapy is a form of ACT, which is composed of extraction of TILs from human tumor samples, *ex vivo* expansion, and reinfusion of expanded autologous lymphocytes into patients following non-myeloablative chemotherapy (9–13). It has been proven an effective anticancer therapy in cervical cancer, ovarian cancer, and particularly in metastatic melanoma (9–13).

Feasibility of TIL expansion from bladder cancer was recently shown by our institution (14). However non-metastatic BC is a heterogenous spectrum of disease ranging from indolent papillary lesions to locally advanced tumors and it represents genomically diverse tumors (15). Proper selection of BC patients with tumors that yield optimal numbers of tumor-reactive TIL has a paramount importance for the success of TIL therapy. In this study, we aimed to evaluate the impact of clinicopathological parameters, molecular subtype (basal vs. luminal) and previous BCG immunotherapy on viable and tumor-reactive TIL expansion.

## Material and Methods

### Patient Selection and Data Collection

Patients older than 18 years of age with previously confirmed pathological diagnosis of BC were included in the study. All patients treated with RC also underwent bilateral pelvic lymph node dissection concurrently. Only patients treated with ≥6 cycles of BCG immunotherapy and completed induction BCG course was considered as BCG-treated. Our previous inclusion criteria was expanded to include patients treated with RC for bladder tumors smaller than 2 cm in size and any bladder lesions/tumors obtained by transurethral resection of bladder tumor (TURBT). The original protocol was amended and approved by the Institutional Review Board (MCC18142). Informed consent was obtained from all patients prior to tissue collection.

### TIL Expansion Protocol

TIL were expanded as previously described (14). Primary bladder tumors or lymph node metastases were minced into ~1–3 mm^3^ fragments and plated in TIL media consisting of RPMI 1640, 2.05 mM L–glutamine (HyClone, Thermo Fisher Scientific, Waltham, MA), 10% heat-inactivated human AB serum (Omega Scientific, Tarzana, CA), 55 μM 2-mercaptoethanol (Invitrogen), 50 μg/ml gentamicin (Invitrogen), 100 I.U./ml penicillin, 100 μg/ml streptomycin, and 10 mM HEPES Buffer (Mediatech, Manassas, VA) in 24- or 48-well plates with 6000 I.U./ml rhIL-2 (Prometheus). Some cultures were supplemented with 1 ug/ml anti-CD137 agonistic antibody (Urelumab, BMS-663513). All cultures were expanded for 4 weeks and confluent wells were split into additional wells. TIL from each independent fragment was counted. Remaining tumor material was mechanically and enzymatically digested using media containing 2% Collagenase Type IV and a GentleMACS Dissociator (Miltenyi, 130–093-235). Cells were counted by trypan blue exclusion and subjected to subsequent analysis or cryopreserved as functional assay targets. Positive TIL growth was defined as confluency and expansion of the primary well into 2 wells.

### Evaluation of TIL Reactivity and Immunophenotyping of Expanded TIL

Flow cytometric analysis of TIL from each fragment was performed. Expanded TIL was stained with fluorescent antibodies for CD3, CD4, CD8, and CD56 (BD Biosciences, BDB555516). All cells were stained with a Live/Dead Near-IR viability stain (Invitrogen, L10119) and fixed in 2% paraformaldehyde. Data were acquired on an LSR II flow cytometer and analyzed using FlowJo software (TreeStar, Inc.). TIL and autologous tumor cells from enzymatic digestion were cultured at a 1:1 ratio (1 x 10^5^ cells each) overnight in round bottom 96-well plates. Supernatants were collected after 24 h. IFN-gamma was measured using a Human IFNg Quantikine ELISA Kit (R&D Systems, SIF50). Optical density of each well was measured at 450 nm and IFN-γ concentration was calculated from the standard curve. IFN-γ concentration ≥100 pg/ml was the cut-off for reactivity.

### Molecular Subtyping

Tumor blocks were retrieved from pathology archive and whole tissue sections were used for immunohistochemistry. Slides were stained using a Ventana Discovery XT automated system (Ventana Medical Systems, Tucson, AZ) as per manufacturer’s protocol with proprietary reagents. Immunohistochemical staining was performed utilizing primary rabbit anti-CD3 antibody (790-4341, Ventana; a predilute concentration), primary rabbit anti-GATA3 antibody (#5852, Cell Signaling Technologies, Danvers, MA; at a 1:200 concentration in Dako antibody diluent, Carpenteria, CA), and mouse monoclonal anti-CK5/6 antibody (790-4554, Ventana; a predilute concentration). The detection system used was the Ventana ChromoMap kit and slides were then counterstained with Hematoxylin. The immunohistochemical results were semi-quantitatively assessed and scored by one pathologist as follows: 0, negative (<1% staining of T cells); 1+, weak (1%–25% staining of T cells); 2+, moderate (26%–64% staining of T cells); and 3+, strong (≥65% staining of T cells).

**Figure 1:**
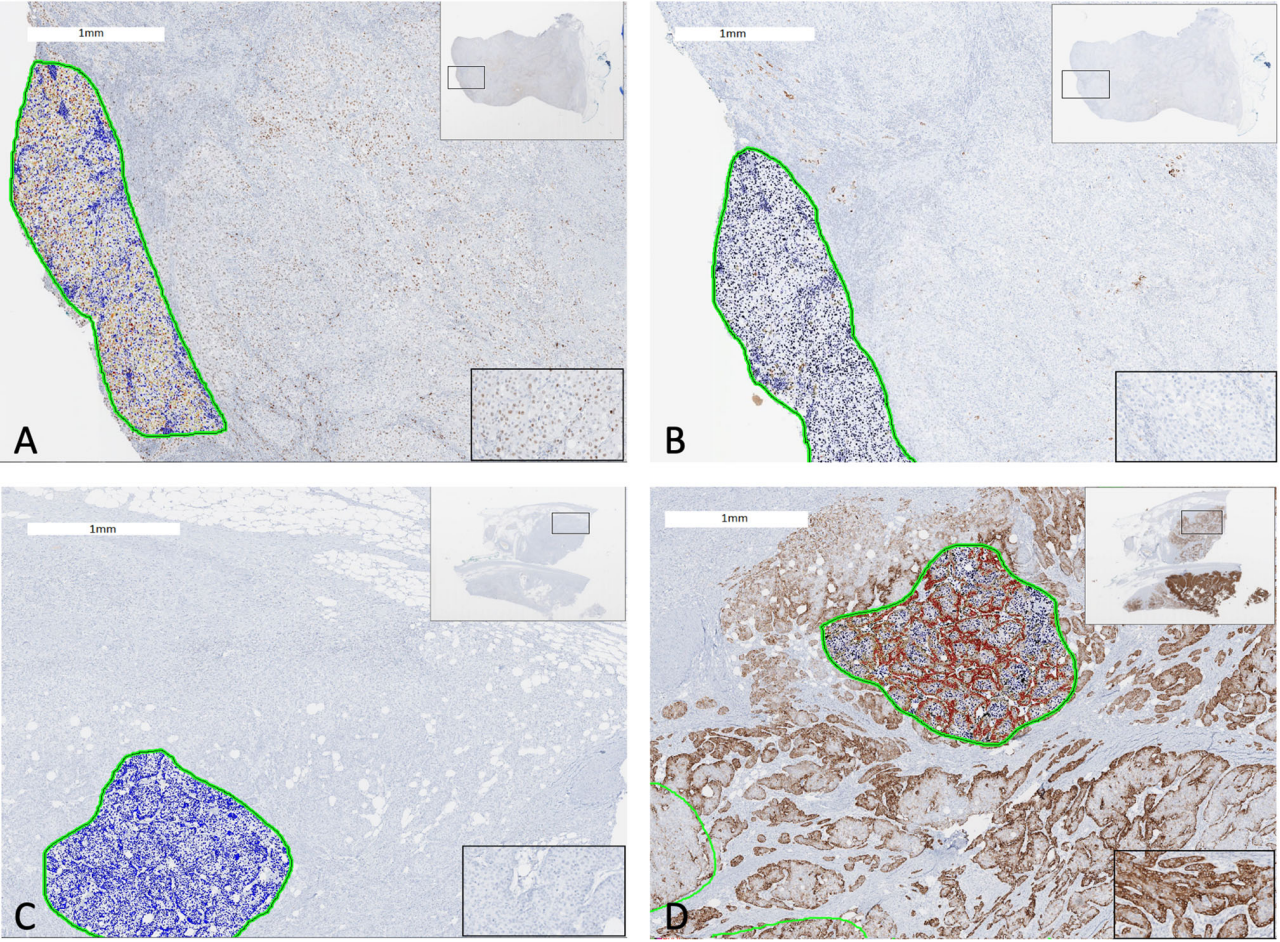
Two cases of bladder cancer showing GATA3 and CK5/6 expression quantified by digital immunohistochemistry analysis. A luminal subtype bladder tumor with **(A)** GATA3 expression ≥20% and **(B)** CK5/6 expression <20% (Left: Anti-GATA3 Ab; Right: Anti-CK5/6 Ab). A basal subtype bladder tumor with **(C)** GATA3 expression <20% and **(D)** CK5/6 expression ≥20% (Left: Anti-GATA3 Ab; Right: Anti-CK5/6 Ab). The green circles represent tumor area analyzed by digital automated immunohistochemistry algorithm. Encircled blue cells/nuclei represents no staining with antibody whereas yellow, orange, brown, dark brown/red ones represent positive staining incremental to staining intensity. Views of whole tumor section at the right upper corner and of a magnified tumor area at the right lower corner.

Molecular characterization of MIBC tumors was performed in primary tumors to identify luminal and basal subtypes *via* immunohistochemistry as previously described by Dadhania et al. (16). Briefly, immunohistochemistry slides stained for GATA3, and CK5/6 were scanned using an Aperio AT2 digital pathology system (Leica Biosystems Inc., Vista, California) with a 20X 0.7NA objective lens. The luminal marker was GATA3 and basal marker was CK5/6. Matching H&E slides were also scanned. Whole slide images were viewed with Aperio Imagescope software and regions of interest (ROIs) were annotated to identify tumor regions. All ROIs were analyzed with Aperio eSlide Manager software using nucleus (GATA3) and membrane (CK5/6) detection algorithms and percent positive values for each sample was calculated. Twenty percent of tumor tissue positivity and 20% of tumor nuclei positivity were cut-off expression levels of the signature basal and luminal markers (**Figure 1**).

### Statistical Analysis

Data represented as scatter plots show individual patient data points as well as error bars representing median values and interquartile range (IQR). The association for categorical and continues variables were evaluated using Fisher’s exact, Chi-square, Mann–Whitney U and Wilcoxon signed rank tests where indicated. The statistical analyses were performed using SPSS version 22.0 (IBM, Armonk, NY) and GraphPad Prism software. p values were two sided and p < 0.05 was considered statistically significant.

## Results

### Patient Characteristics

A total of 51 primary tumors and 17 additional LN from 53 patients were included for the analysis of factors that are associated with overall TIL expansion as well as expansion of reactive TIL (**Table 1**). Briefly, 43 (81.1%) patients underwent RC while 10 (18.9%) patients underwent TURBT. Eleven samples (20.8%) were collected from patients who had received intravesical BCG immunotherapy and 22 (41.5%) samples were collected from patients who had received NAC. Among 40 (71.4%) patients with MIBC, 10 patients were found to have basal tumors whereas 25 patients had luminal tumors.

**Table 1:** Characteristics of 53 bladder cancer patients included in the analysis.

Variable	Patient number (%)
Age	
<60	8 (15.1)
60–69	16 (30.2)
70–79	21 (39.6)
≥80	8 (15.1)
Gender	
Female	14 (26.4)
Male	39 (73.6)
Ethnicity	
Non-hispanic white	51 (96.2)
Other	2 (3.8)
BMI	
18.5-24.9	17 (32.1)
25.0-29.9	17 (32.1)
≥30.0	15 (28.3)
Unknown	4 (7.5)
Surgery	
Radical cystectomy	43 (81.1)
Transurethral resection	10 (18.9)
Smoking status	
Never smoker	11 (20.7)
Ever smoker	32 (60.4)
Current smoker	10 (18.9)
pT stage	
Ta/Tis/T1	13 (24.5)
T2	15 (28.3)
T3	15 (28.3)
T4	10 (18.9)
pN stage (MIBC)	
N0	29 (67.5)
N1	6 (13.9)
N2	2 (4.7)
N3	6 (13.9)
Variant histology	
Pure	29 (54.7)
Mixed	24 (45.3)
Type of histology	
Pure urothelial carcinoma	29 (54.7)
Squamous differentiation	11 (20.8)
Plasmacytoid	4 (7.5)
Micropapillary	3 (5.7)
Sarcomatoid	2 (3.8)
Other variants^a^	4 (7.5)
Molecular subtype of MIBC	
Basal	10 (25.0)
Luminal	25 (62.5)
N/A	5 (12.5)
Prior BCG immunotherapy	11 (20.8)
Prior neoadjuvant chemotherapy	22 (41.5)

BCG, Bacillus Calmette-Guerin; BMI, body mass index; MIBC, muscle-invasive bladder cancer; pN, pathological lymph node; pT, pathological tumor; Tis, carcinoma in-situ; TIL, tumor-infiltrating lymphocytes; N/A, not applicable/evaluable.

a One glandular differentiation, one microcystic, one clear cell, and one squamous cell carcinoma.

### TIL Growth and Reactivity

In our previous study, we showed TIL expansion from 70% of primary bladder tumors as well as TIL expansion from all LNs (20 primary tumors and 7 LN from 20 patients) (14). In this study, we collected tumor fragments from an additional 33 patients (31 primary tumors and 10 LN) and confirmed that TIL growth could be achieved in 37 out of 53 (69.8%) patients using primary tumors and/or LN (**Table 2**). In this study, autologous tumor was available for TIL samples in 29 patients. Co-culture of autologous tumor with expanded TIL was performed and production of IFN-gamma was measured. Expanded TIL from primary tumors and/or LNs showed anti-tumor reactivity in 20 out of 29 (69.0%) patients, thus overall reactivity of expanded TIL per patient was 74.3% (26/35 patients; six patients from the previous study).

**Table 2:** Expansion of tumor-reactive TIL in 53 bladder cancer patients.

Variable	Number
Overall TIL growth per patient	
Yes	37 (69.8%)
No	16 (30.2%)
TIL growth from PT	
Yes	33 (64.7%)
No	18 (35.3%)
Median TIL number from PT ^a^	1.86E+07 (1.60E+06 - 3.09E+08)
TIL growth from LN	
Yes	16 (94.1%)
No	1 (5.9%)
Median TIL number from LN ^b^	4.73E+07 (4.40E+06 - 2.86E+08))
Overall reactivity of TIL per patient	
Yes	26 (74.3%)
No	9 (25.7%)
N/A	2
Reactivity of TIL from PT	
Yes	18 (60.0%)
No	12 (40.0%)
N/A	3
Reactivity of TIL from LN	
Yes	10 (83.4%)
No	2 (16.6%)
N/A	4

LN, lymph node; PT, primary tumor; TIL, tumor-infiltrating lymphocytes; N/A, not applicable/evaluable.

a Median (range) number of TIL that grew from 33 primary tumor fragments.

b Median (range) number of TIL grew from 16 lymph node fragments.

TIL growth was achieved from additional nine out of 10 LN samples in the present study, thus the rate of TIL growth from LN samples was 94.1% (16/17 LNs; 7 LNs from the previous study). Co-culture with autologous tumor was feasible in 12 expanded TIL from LNs, and 10 expanded TIL showed anti-tumor reactivity (83.4%). The median number of expanded TIL from primary tumors and LN was 1.86E+07 and 4.73E+07 in 33 and 16 patients, respectively.

### Factors Associated With Expansion and Anti-Tumor Reactivity of TIL

We next evaluated the factors associated with TIL expansion and tumor-reactivity of TIL among a total of 53 patients (20 patients from previous study and additional 33 patients). TIL growth was feasible across all patient subgroups (**Table 3**). The number of expanded TIL from primary tumors and LN were similar between patients regardless of age, gender, smoking status, BMI, type of surgery, pathological tumor/LN stage, histology, molecular subtype, previous NAC and weight of fragment (data now shown) with the exception of previous BCG immunotherapy.

**Table 3:** Factors affecting growth and reactivity of TIL harvested from primary tumors and/or lymph nodes in 53 patients.

Variable	TIL Growth			TIL Reactivity^1^		
	No, n(%)	Yes, n(%)	P value	No, n(%)	Yes, n(%)	P value
Patients	16 (30.2)	37(69.8)	–	9 (25.7)	26 (74.3)	–
Age, yr.^2^	72.5 [57.6;88.4]	70.0 [51.8;91.9]	0.461	77.0 [65.0;90.5]	68.5 [51.8;91.9]	0.026
Gender			0.510			0.685
Male	13 (33.3)	26 (66.7)		7 (29.2)	17 (70.8)	
Female	3 (21.4)	11 (78.6)		2 (18.1)	9 (81.9)	
BMI^2^	26.1 [20.1;36.7]	26.8 [19.2;49.2]	0.834	26.8 [19.2;36.8]	27.6 [19.7;49.2]	0.558
Smoking status			0.470			0.191
Never	2 (18.2)	9 (81.8)		4 (44.4)	5 (55.6)	
Ever/Current	14 (33.3)	28 (66.7)		5 (19.2)	21 (80.8)	
Type of surgery			0.050			0.267
Radical cystectomy	10 (23.3)	33 (76.7)		7 (22.6)	24 (77.4)	
TURBT	6 (60.0)	4 (40.0)		2 (50.0)	2 (50.0)	
pT stage			0.234			0.353
Ta/Tis/T1	6 (46.2)	7 (53.8)		3 (42.8)	4 (57.2)	
T2	5 (33.3)	10 (66.7)		3 (30.0)	7 (70.0)	
T3/4	5 (20.0)	20 (80.0)		3 (16.6)	15 (83.4)	
pN stage (MIBC)			0.703			1.000
N0	6 (20.7)	23 (79.3)		5 (21.7)	18 (78.3)	
N+	4 (28.6)	10 (71.4)		2 (25.0)	6 (75.0)	
Variant histology			0.073			0.049
Pure	12 (41.4)	17 (58.6)		7 (43.7)	9 (56.3)	
Mixed	4 (16.6)	20 (83.4)		2 (10.5)	17 (89.5)	
Molecular subtype^3^			0.999			0.130
Basal	2 (20.0)	8 (80.0)		0	8 (100.0)	
Luminal	7 (28.0)	18 (72.0)		5 (31.2)	11 (68.8)	
Prior NAC			0.768			1.000
No Yes	10 (31.2) 6 (29.1)	21 (68.8) 16 (70.9)		5 (26.3) 4 (25.0)	14 (73.7) 12 (75.0)	
Prior intravesical BCG			0.716			0.027
No	12 (37.5)	30 (62.5)		5 (17.4)	24 (82.6)	
Yes	4 (36.4)	7 (63.6)		4 (66.7)	2 (33.3)	
Tumor weight, gr.^2^	0.60 [0.17;8.56]	1.17 [0.04;5.06]	0.150	0.66 [0.34;1.64]	1.48 [0.34;5.06]	0.102
Sample used^4^			0.026			0.276
Primary tumor	18 (35.3)	33 (64.7)		12 (40.0)	18 (60.0)	
Lymph node	1 (5.5)	16 (94.1)		2 (16.6)	10 (83.4)	

BCG, Bacillus Calmette-Guerin; BMI, Body mass index; MIBC, muscle-invasive bladder cancer; NAC, Neoadjuvant chemotherapy; Tis, carcinoma in-situ; pN, pathological lymph node; pT, pathological tumor; TIL, tumor-infiltrating lymphocytes; TURBT, Transurethral resection of bladder tumor

^1^TIL reactivity could not be measured due to lack of autologous tumor in 2 patients.

^2^Values are presented as median and range (min; max).

^3^Molecular subtyping was performed for MIBC. Tissue was not available in 5 patients with MIBC.

^4^Autologous tumor was not available for measurement of reactivity for 3 TIL samples grown from primary tumor and 4 TIL samples grown from lymph node.

Anti-tumor reactivity of expanded TIL was lower in primary tumors treated with BCG immunotherapy compared to those without (33.3% vs. 82.6%, p= 0.027). Other significant finding of the reactivity analysis included the age of patients and tumor histology. The median age of patients yielding tumor reactive TIL was significantly younger than the median age of patients with expanded TIL showing no anti-tumor reactivity (68.5 [51.8;91.9] vs. 77.0 [65.0;90.5], p=0.026). However the surgical specimen from the oldest patient in our cohort yielded higher amounts of reactive TIL (4.06E+07). Thus, age might not be a limiting factor for expansion of TIL although it warrants further investigation with additional specimens.

TIL reactivity from mixed urothelial tumors was significantly higher than pure urothelial carcinoma (89.5% vs. 56.3%, p=0.049). Basal subtype tumors yielded higher TIL growth (80%) with more anti- tumor reactivity (100%) compared to luminal subtype (72% TIL growth and 68.8% tumor-reactive TIL) albeit statistically not significant (p=0.999 and p=0.130). Thus, we identified a subset of BC tumors (mixed histology and BCG-naïve tumors) with enhanced TIL reactivity.

### Previous BCG Immunotherapy and Tumor-Reactive TIL Expansion

**Figure 2:**
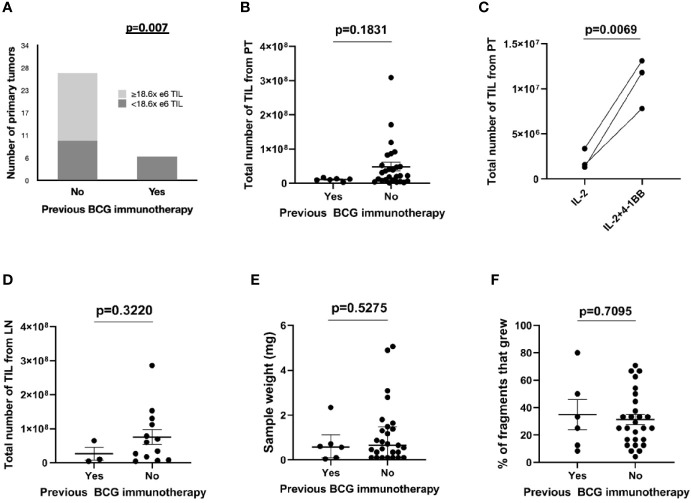
Expansion of TIL from bladder tumors stratified by BCG immunotherapy. The total number of TIL expanded from bladder or LN tumor fragments was measured at 4 weeks after initiation of culture. **(A)** TIL growth from primary tumors stratified by previous BCG immunotherapy and median number of expanded TIL in 33 patients. Fisher’s exact test. **(B)** Total number of expanded TIL from BCG-naïve primary tumors (27 patients) and from primary tumors with previous exposure to BCG immunotherapy (six patients). Each point represents the total TIL per patient (the sum of all TIL that was generated from each fragment within an individual patient). Median and interquartile range. Mann-Whitney test. **(C)** Total numbers of expanded TIL from three primary tumors previously treated with BCG immunotherapy. Fragments of the surgical specimens were cultured either in IL-2 (6000 IU/ml) only or in IL-2 (6000 IU/ml) + 4-1BB (10 ug/ml). Wilcoxon-signed rank test. **(D)** Total number of expanded TIL from BCG-naïve lymph nodes (13 patients) and from lymph nodes with previous exposure to BCG immunotherapy (three patients). Each point represents the total TIL per patient (the sum of all TIL that was generated from each fragment within an individual patient). Median and interquartile range. Mann-Whitney test. **(E)** The sample weight (mg) of primary tumors sent to the lab for expansion of TIL (six BCG-treated samples and 27 BCG-naive samples). Median and interquartile range. Mann–Whitney test. **(F)** The percentage of fragments that grew TIL from the total number of fragments plated (six samples from BCG-treated primary tumors and 27 samples from BCG-naive primary tumors). Median and interquartile range. Mann–Whitney test.

**Figure 3:**
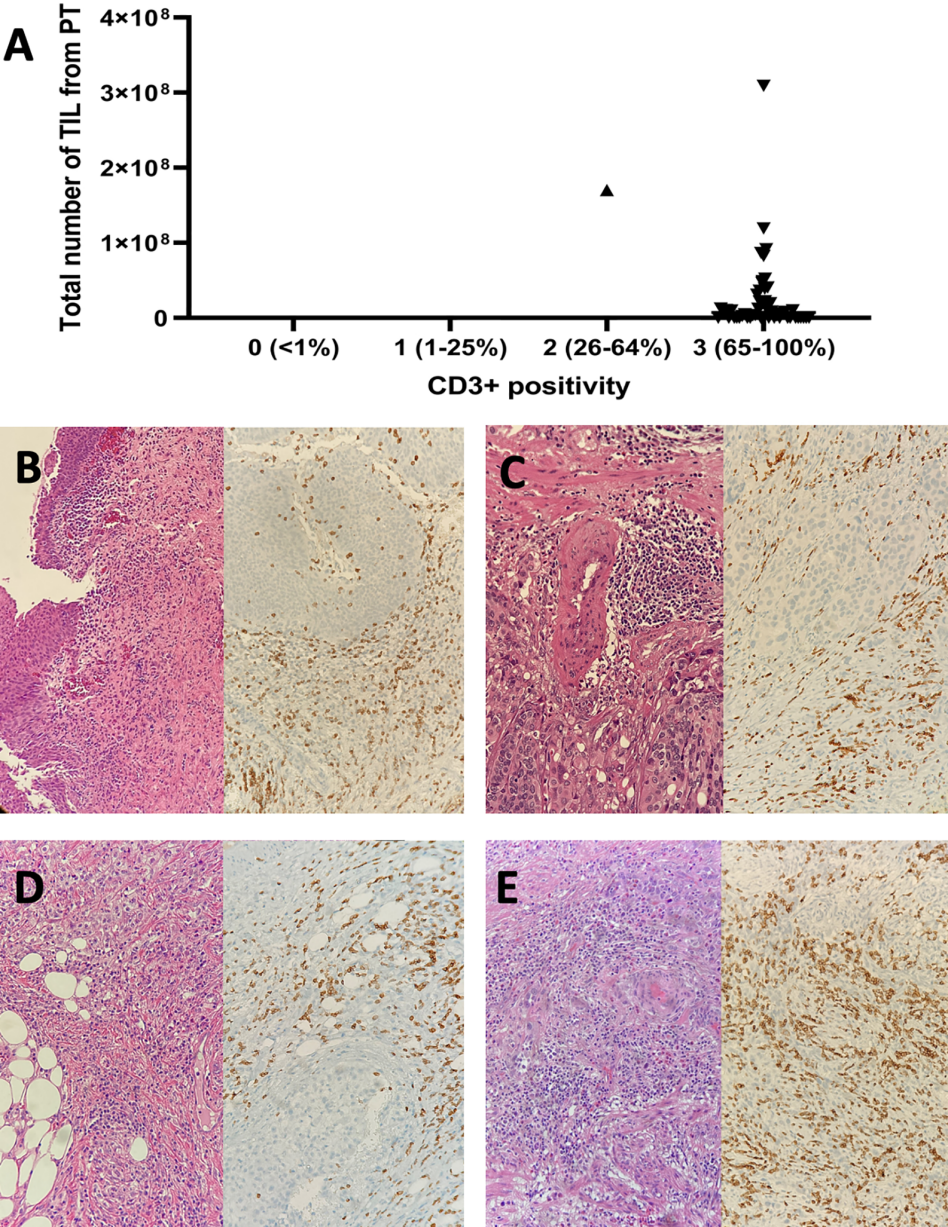
The CD3+ tumor infiltrating lymphocyte infiltration within tumor microenvironment and expansion of TIL from primary bladder tumors. **(A)** Expansion of TIL was achieved in 33 out of 51 primary tumors, of which 50 and one displayed strong and moderate CD3+ T cell infiltration, respectively. **(B)** A pTis bladder tumor (pure urothelial call carcinoma) previously treated with BCG immunotherapy (Left: H-E x 100; Right: Anti-CD3 Ab x 200). **(C)** A pT2 bladder tumor (pure urothelial cell carcinoma) previously treated with BCG immunotherapy (Left: H-E x 200; Right: Anti-CD3 Ab x 200). **(D)** A pT3 bladder tumor (pure urothelial carcinoma) with no previous exposure to intravesical BCG (Left: H-E x 200; Right: Anti-CD3 Ab x 200). **(E)** A pT2 bladder tumor (squamous differentiation) with no previous exposure to intravesical BCG (Left: H-E x 200; Right: Anti-CD3 Ab x 200). H-E, hematoxylin and eosin; Ab, antibody; pT, pathological tumor stage.

**Figure 4:**
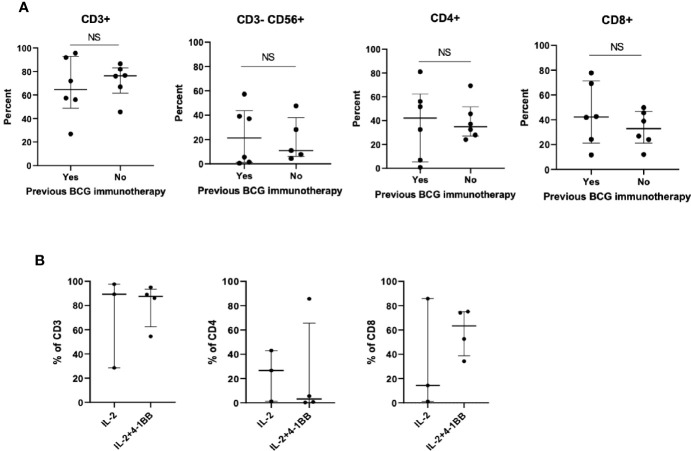
Phenotype of TIL expanded from primary bladder tumors previously treated with BCG immunotherapy. **(A)** Six BCG-treated patients whose primary bladder tumors yielded TIL were matched with six BCG-untreated patients (1:1) in terms of patient age, tumor histology, tumor stage and molecular subtype of bladder cancer. At 4 weeks after the initiation of TIL cultures, TIL were collected from each fragment and the percentage of CD3+ T cells, CD4+ T cells, CD8+ T cells, and CD3-CD56+ NK cells was measured by flow cytometry. Each point represents the mean percentage of cells per patient (the mean of all TIL that was generated from each fragment within an individual patient). Wilcoxon-signed rank test. **(B)** Tumor fragments of three primary tumors previously treated with BCG immunotherapy were cultured either in IL-2 (6000 IU/ml) + 4-1BB (10 ug/ml) or in only IL-2 (6000 IU/ml). Percentage of CD3+ T cells, CD4+ T cells, and CD8+ T cells was measured by flow cytometry. Each point represents the percentage of cells generated from each tumor fragment. Median and interquartile range.

The number of expanded TIL was lower than overall median TIL number (1.86E+07) in all (6/6) primary tumors previously treated with BCG compared to those from primary tumors without previous BCG exposure (10/27 (37.0%), p=0.007, **Figure 2**). This was not due to a lack of T cells in tumors since all primary tumors displayed moderate to strong infiltration of CD3+ tumor-infiltrating lymphocytes (scores ≥2+) regardless of history of BCG immunotherapy (**Figure 3**). The sample weight, and the percent of tumor fragments that grew TIL were also comparable between each group. To attempt to improve TIL expansion in these cultures, we added agonistic anti-4-1BB antibodies to culture media as it was previously found to enhance the expansion of TIL in malignant melanoma and bladder cancer treated with NAC by our institution (14, 17). Tumor fragments of three primary tumors were cultured in IL-2 (6000 IU/ml) + anti-4-1BB antibody (10 ug/ml) and these fragments yielded higher numbers of expanded TIL compared to fragments of the same tumors cultured only in IL-2 (6000 IU/ml) (p=0.006). There were no significant difference in composition of CD3+, CD4+, CD8+, and CD56+ T cells in expanded TIL regardless of BCG exposure or agonism with anti-4-1BB antibodies (**Figure 4**).

## Discussion

BC is morphologically and genomically a heterogenous malignancy and unique owing to utilization of immunotherapy for treatment of both early and late stage tumors (18). Moreover, BC has the third highest tumor mutational burden among all cancer types, which was associated with high neoantigen load and enrichment of activated immune cells within tumor site (19). Recently, our group demonstrated that TIL expansion from BC was feasible (70%) in a homogenous cohort of 20 patients treated with RC for bladder tumors larger than 2 cm (14). In the present study, we included an additional 33 patients, including patients who had smaller RC tumors (<2 cm) and patients who were treated with TURBT. We also measured anti-tumor reactivity of all expanded TIL if autologous tumors were available. In this expanded cohort of patients with BC, the success of viable TIL growth was 69.8%, which was consistent with our preliminary report (70%) and those reported for patients with malignant melanoma (ranging between 75% and 85%) (20). Moreover, we were able to utilize metastatic LN sent together with primary tumors in RC specimens for TIL expansion. Overall, TIL growth was feasible across all subgroups of BC patients. The rate of reactivity of expanded TIL against autologous tumors was 74.3% in our study, comparable to that reported for malignant melanoma (67%) (21).

Overall TIL reactivity from tumor samples of variant histology or squamous/glandular differentiation (mixed histology) was significantly higher than that of pure urothelial carcinoma (89.5% vs. 56.3%). Presence of variant histology in NMIBC is considered as a high risk feature for progression to a higher stage disease and early RC is recommended for these cases (18). The therapeutic options for MIBC with variant histology is limited and response to NAC is modest without increase in overall survival (22). In contrast, preliminary reports suggest a substantial benefit from immunotherapy for MIBC with variant histology, downstaging to pT1 or less was achieved after neoadjuvant ICB in about half of the patients harboring such muscle-invasive disease (23). Particular variants of urothelial cell carcinoma have unique gene expression profiles and some characterized by enrichment with genomic signatures predictive for immunotherapy response or high PD-L1 expression (23–25). Based on anti-tumor reactivity of expanded TIL from mixed histology tumors in our study, we suggest that TIL therapy might have a potential to address the unmet need of perioperative systemic therapy for these tumors.

Interestingly, anti-tumor reactivity of expanded TIL from primary tumors previously treated with BCG immunotherapy was lower than those without (33% vs. 83%). Likewise, the number of expanded TIL appeared to be lower from such tumors. Of note, the number of BCG cycles and the interval time between last BCG instillation and TIL expansion varied across the patients, however additional maintenance BCG was not found to further decrease TIL reactivity after induction BCG (data not shown). The decrease in anti-tumor reactivity might be due to BCG-related indirect mechanisms mediated by other components of tumor microenvironment such as MDSCs, regulatory T cells or increased expression of particular cytokines after exposure to BCG. Likewise, Chevalier et al. reported lower T cell-to-monocytic myeloid-derived suppressor cells (MDSC) ratios, higher frequencies of group 2 innate lymphoid cells, and detectable levels of IL-13, an inducer for suppressive functions in monocytes, in the urine of patients who failed intravesical BCG (26). Whether BCG exhausted TIL in the tumor microenvironment prior to harvesting was not clear and it warrants further investigation. However, the overall CD3+ T cell concentrations in primary tumors appeared similar across the patients in our study. The percentages of CD4+, CD8+, and CD3-CD+56 T cells in expanded TILs appeared to be not effected by previous BCG therapy, consistent with animal studies evaluating effect of BCG on T cell phenotype (27). Moreover, we could effectively multiply the number of expanded TIL from BCG treated primary tumors with agonistic anti-4-1BB. Thus, the effect of previous BCG immunotherapy on tumor-reactive TIL expansion warrants further investigation.

Unsurprisingly, an accurate predictive biomarker for expansion of viable and reactive TIL is invaluable. Particular molecular subtypes of BC were found to have predictive value and associate with significant immunological signatures (28). Basal subtype bladder tumors were associated with higher infiltration of CD8+ T cells and NK cells, advanced disease stage and lower median overall survival compared to luminal subtype tumors (1.2 years vs. 1.8–4.0 years). Patients with basal subtype tumors with high RNA-based immune signature had a 100% 2-year progression-free survival after neoadjuvant pembrolizumab therapy in a phase II MIBC trial (29). In our study, we did not observe any significant association between molecular subtype and viable and reactive TIL growth although basal subtype bladder tumors appeared to yield more reactive TIL, which warrants further investigation. For molecular subtyping, we used a validated immunohistochemistry method and quantitative scoring to improve reproducibility. Although basal tumors were shown to be almost exclusively basal, luminal subtypes appeared to consist of a more heterogenous group of tumors (16). A comprehensive genomic analysis would enable identification of other molecular subtypes such as luminal-papillary, luminal-unstable and neuroendocrine-like and a more reproducible molecular subtype profiling (28). Nonetheless, the value of molecular characterization as a predictive biomarker in BC appears to be limited due to significant tumor heterogeneity and impact of previous treatments (30). TCGA molecular subtype did not appear to be a strong predictor of response to Atezolizumab in patients with refractory metastatic urothelial cancer (31). We are currently exploring the diversity of T cell populations and neoantigen-specific T cells in expanded TIL from bladder tumors to identify predictive biomarkers for TIL expansion. Moreover, reliable identification of metastatic LNs in the operating room, further optimization of TIL culture conditions, and comprehensive *in vitro* measurement of anti-tumoral T cell activity can further increase the success of tumor-reactive TIL expansion, which warrant further investigation.

The observed success in TIL expansion increases the potential to implement clinical trial of TIL therapy for BC patients in the near future. Conventional TIL therapy strategies focusing on treatment of metastatic cancer consists of systemic infusion of expanded TIL in patients preconditioned with non-myeloablative chemotherapy (such as cyclophosphamide and fludarabine) (32, 33). We demonstrated that expansion of tumor-reactive TIL is feasible from bladder tumor samples obtained *via* TURBT. TIL therapy can potentially be a novel treatment option in the non-metastatic setting including patients with BCG-naïve NMIBC. Clinical trials of intravesical TIL therapy with or without intravesical IL-2, alone or in combination with systemic PD-1 inhibitor or intravesical BCG appears reasonable. Acknowledging our imperfect ability to detect all tumor recognition *in vitro*, the polyclonal nature of TIL therapy, and demonstrated feasibility of TIL growth from BCG-treated tumor samples, a subset of BCG-unresponsive NMIBC may also benefit from TIL therapy.

## Conclusions

Tumor fragments of both primary tumor and LN can yield TIL with tumor-specific reactivity in BC patients. Specifically, we identified a subset of BC patients with improved TIL reactivity including patients with mixed histology and BCG-naïve tumors. Nonetheless, TIL therapy appears to be feasible for any patient with BC, and our findings suggest feasibility for TIL clinical trials for management of BC.

## Data Availability Statement

The raw data supporting the conclusions of this article will be made available by the authors, without undue reservation.

## Ethics Statement

The studies involving human participants were reviewed and approved by the Institutional Review Board (MCC18142). The patients/participants provided their written informed consent to participate in this study.

## Author Contributions

AA, BB, MB, SP-T, and MP designed the study. AA and MP procured the biological specimen. JD was in charge of the pathology. AA, BB, MB, and SP-T performed the experimental work. AA, BB, MB, SP-T, and MP analyzed and interpreted the data. All authors contributed to the article and approved the submitted version.

## Funding

This work was funded in part by Iovance Biotherapeutics, Swim Across America, and the Dr. Miriam and Sheldon G. Adelson Medical Research Foundation. AS was supported by NCI-5K23CA178083. SP-T was supported by an American Cancer Society—Leo and Anne Albert Charitable Foundation Research Scholar Grant (RSG-16-117-01-LIB). The funder bodies were not involved in the study design, collection, analysis, interpretation of data, the writing of this article or the decision to submit it for publication.

## Conflict of Interest

Moffitt Cancer Center has licensed Intellectual Property (IP) related to the proliferation and expansion of tumor infiltrating lymphocytes (TILs) to Iovance Biotherapeutics. SP-T and AS are inventors on such Intellectual Property. SP-T and AS are listed as co-inventors on a patent application with Provectus Biopharmaceuticals. Moffitt has also licensed IP to Tuhura Biopharma, and SP-T is an inventor on such Intellectual Property.

The remaining authors declare that the research was conducted in the absence of any commercial or financial relationships that could be construed as a potential conflict of interest.
